# Complete Mitochondrial Genome Sequence of a Seabird, Wedge-Tailed Shearwater (Ardenna pacifica)

**DOI:** 10.1128/mra.01191-21

**Published:** 2022-02-10

**Authors:** Subir Sarker, Md. Hakimul Haque, Babu Kanti Nath, Saranika Talukder, Jennifer L. Lavers, Shane R. Raidal

**Affiliations:** a Department of Microbiology, Anatomy, Physiology and Pharmacology, School of Agriculture, Biomedicine and Environment, La Trobe University, Melbourne, Victoria, Australia; b Department of Veterinary and Animal Sciences, Faculty of Agriculture, Rajshahi University, Rajshahi, Bangladesh; c School of Animal and Veterinary Sciences, Faculty of Science, Charles Sturt University, Bathurst, New South Wales, Australia; d School of Agriculture and Food, Faculty of Veterinary and Agricultural Sciences, The University of Melbourne, Melbourne, Victoria, Australia; e Institute for Marine and Antarctic Studies, University of Tasmania, Hobart, Tasmania, Australia; Vanderbilt University

## Abstract

Here, we report the complete mitochondrial genome sequence of a seabird, wedge-tailed shearwater (Ardenna pacifica). The circular genome has a size of 16,434 bp and contains 13 protein-coding genes, 22 tRNA genes, and 2 rRNA genes. The study provides a reference mitochondrial genome of wedge-tailed shearwater for further molecular studies.

## ANNOUNCEMENT

The genus *Ardenna* in the family Procellariidae comprises seven species of seabirds. Among them, the wedge-tailed shearwater (Ardenna pacifica) is a colonial, burrow-nesting petrel with an extensive distribution in the tropical and subtropical waters of the Indian and Pacific Oceans (1, 2). Molecular-method-based studies on A. pacifica are minimal, and only partial mitochondrial sequences of this species are available in GenBank (3, 4). A study used nucleotide sequences of cytochrome *b* genes to better understand the phylogeny and taxonomy of the species in the order Procellariiformes and suggested that the data were insufficient to resolve all higher relationship issues, which warrants additional mitochondrial DNA and nuclear sequence data analysis (4). To rectify these limitations, the present study aimed to sequence a mitochondrial genome of A. pacifica.

A cutaneous tissue sample (sample identification number CS15-1526) was collected from a wedge-tailed shearwater (Ardenna pacifica) originating from Lord Howe Island, New South Wales, Australia (32.53°S, 159.08°E), and was deposited at Charles Sturt University. Animal sampling was conducted with the permission of the Lord Howe Island Board (permit number LHIB 02/14) and the approval of the University of Tasmania and Charles Sturt University animal ethics committees (permit numbers A0010874, A0011586, and 09/046). The DNA was isolated using the DNeasy blood and tissue kit (Qiagen, Germany) according to the manufacturer's guidelines (5, 6). The library was prepared using the TruSeq DNA library preparation kit v2 (Illumina, San Diego, CA) and sequenced using the Illumina HiSeq 4000 sequencing platform, generating 150-bp paired-end reads (5, 7). Sequencing data were analyzed with an established pipeline (5, 7–9) using Geneious (v10.2.2; Biomatters, New Zealand) and CLC Genomics Workbench (v9.5.4). Briefly, a total of 15.36 million raw reads were preprocessed to remove the Illumina adapters, ambiguous base calls, and poor-quality reads (trim using quality score, limit 0.05; trim ambiguous nucleotide up to 15 using CLC Genomics Workbench), followed by mapping against an Escherichia coli bacterial genomic sequence (GenBank accession number U00096) to eliminate possible bacterial contamination. Trimmed and unmapped clean reads were used as input data for *de novo* assembly in CLC Genomics Workbench (v9.5.4) with default parameters. This resulted in the generation of a 16,434-bp mitogenome obtained from A. pacifica. A total of 14.10 million clean reads were mapped back to the mitogenome of A. pacifica, which resulted in an average coverage of 91.60×. The final mitogenome of A. pacifica was circularized using Geneious software with default parameters. Annotation of the assembled mitogenome of A. pacifica was performed using default parameters with the Vertebrate Mitochondrial Code (transl_table=2) in Geneious (v10.2.2).

**FIG 1:**
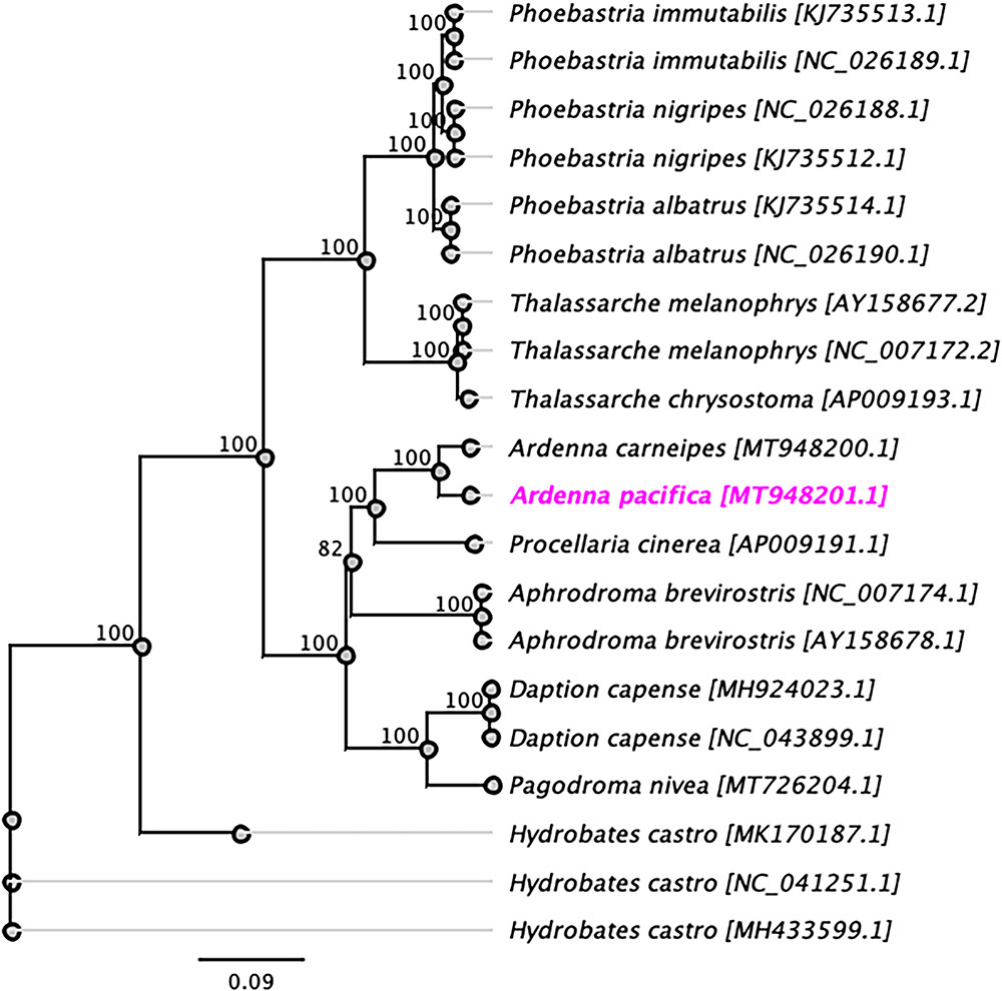
Maximum likelihood phylogenetic tree to infer host phylogenetic relationships using the mitochondrial genome sequenced from A. pacifica along with other selected species in the order Procellariiformes. The nucleotide sequences were aligned with MAFFT (v7.450) using the G-INS-i algorithm (gap open penalty, 1.53; offset value, 0.123) implemented in Geneious (v7.388) (10). A phylogenetic tree was constructed under the GTR substitution model in Geneious (v10.2.2) with 500 bootstrap replicates. The new complete mitogenome of A. pacifica is highlighted in bold and magenta. Labels at the branch tips refer to species, followed by GenBank accession numbers in brackets.

The assembled complete mitogenome of the wedge-tailed shearwater had a circular genome of 16,434 bp, with a G+C content of 43.5%. Our annotation identified 1 control region (D-loop), 2 rRNA coding regions (12S and 16S rRNAs), 22 tRNAs, and 13 protein-coding genes. Phylogenetic analysis using complete mitogenome sequences showed that A. pacifica was grouped into a well-supported subclade with the flesh-footed shearwater (Ardenna carneipes [GenBank accession number MT948200]) (7) (Fig. 1) and demonstrated 95.61% pairwise nucleotide identity between them. We concluded that the complete mitogenome of A. pacifica will be useful data for the genus *Ardenna* to study the host phylogenetic relationships further.

### Data availability.

The complete mitochondrial genome sequence of A. pacifica has been deposited in DDBJ/ENA/GenBank under the accession number MT948201. The version described in this paper is the first version, MT948201.1. The raw sequencing data from this study have been deposited in the NCBI Sequence Read Archive (SRA) under the accession number SRR14626654 (BioProject accession number PRJNA732113).
